# Robust and Task-Independent Spatial Profile of the Visual Word Form Activation in Fusiform Cortex

**DOI:** 10.1371/journal.pone.0026310

**Published:** 2011-10-14

**Authors:** Lifei Ma, Yi Jiang, Jian'e Bai, Qiyong Gong, Haicheng Liu, Hsuan-Chih Chen, Sheng He, Xuchu Weng

**Affiliations:** 1 Key Laboratory of Mental Health, Institute of Psychology, Chinese Academy of Sciences, Beijing, China; 2 Department of Psychology, University of Minnesota, Minneapolis, Minnesota, United States of America; 3 Huaxi MR Research Center (HMRRC), Department of Radiology, West China Hospital of Sichuan University, Chengdu, China; 4 Department of Psychology, Chinese University of Hong Kong, Hong Kong S.A.R., China; 5 Center for Cognition and Brain Disorders, Hangzhou Normal University, Hangzhou, Zhejiang, China; Beijing Normal University, Beijing, China

## Abstract

Written language represents a special category of visual information. There is strong evidence for the existence of a cortical region in ventral occipitotemporal cortex for processing the visual form of written words. However, due to inconsistent findings obtained with different tasks, the level of specialization and selectivity of this so called visual word form area (VWFA) remains debated. In this study, we examined category selectivity for Chinese characters, a non-alphabetic script, in native Chinese readers. In contrast to traditional approaches of examining response levels in a restricted predefined region of interest (ROI), a detailed distribution of the BOLD signal across the mid-fusiform cortical surface and the spatial patterns of responses to Chinese characters were obtained. Results show that a region tuned for Chinese characters could be consistently found in the lateral part of the left fusiform gyrus in Chinese readers, and this spatial pattern of selectivity for written words was not influenced by top-down tasks such as phonological or semantic modulations. These results provide strong support for the robust spatial coding of category selective response in the mid-fusiform cortex, and demonstrate the utility of the spatial distribution analysis as a more meaningful approach to examine functional magnetic resonance imaging (fMRI) data.

## Introduction

A number of recent studies have suggested that an area in the left mid-fusiform region near the occipital-temporal sulcus may be specialized for the processing of written text. Indeed, some researchers have labeled this area the Visual Word Form Area (VWFA) [1], [2], [3], [4]. The response of this area is invariant to the stimulus location, and independent of word font, size and case [1]. This area is consistently activated during word reading in normal readers [5], [6], [7], [8], and activity in this region was even shown to be causally related to the word recognition of a patient who underwent surgical removal of a portion of occipito-temporal cortex overlapping with the presumed VWFA[9].

However, there remains a lack of consensus on the level of selectivity (e.g., words or letter strings, see [10], [11], [12], [13]. Indeed, others have questioned the characterization of this area's functional properties, arguing instead that this region is responsive to many more categories of visual stimuli [14], [15], [16], [17], or may serve as a link between visual analysis and high level phonological and semantic representation [18], [19], [20], [21], [22], [23], and may not be specific to the visual modality [24].

We believe there are two interlinked factors that contribute to the dispute over the specialization of this region. First, the identification of an ROI critically depends on the control condition as well as the statistical threshold. Variations in the control condition and threshold lead to inconsistent identification of the key ROI (i.e., VWFA) across studies. Second, the response amplitude in a predefined ROI (e.g., VWFA) could be modulated by many factors including task relevance and attentional load. Thus a variable degree of top-down influence (e.g., differential level of semantic/phonological processing) may contribute to the inconsistent results regarding the level of category selectivity of the VWFA, even at high resolution [25].

The current study aimed to characterize the detailed representation of Chinese characters in mid-fusiform cortex in terms of the spatial profile of activity (Exp. 1), which highlights the invariant spatial aspects of category-specific response selectivity. Further, the study also examined the distribution of activation under different task demands (Exp. 2).

## Materials and Methods

### Ethics Statement

All experiments and procedures were approved by the IRB of the Institute of Psychology, Chinese Academy of Sciences and the IRB of the University of Minnesota. All participants gave informed consent before taking part in the experiments.

### Participants

Eleven healthy volunteers (native Chinese readers who have also extensive English reading experience) with a high education took part in Experiment 1. Volunteers were aged between 21–32 years (mean = 25±2.4). Another seven native Chinese readers took part in Experiment 2. All participants were right-handed and had normal or corrected-to-normal vision.

### Stimuli and Procedure

**Figure 1:**
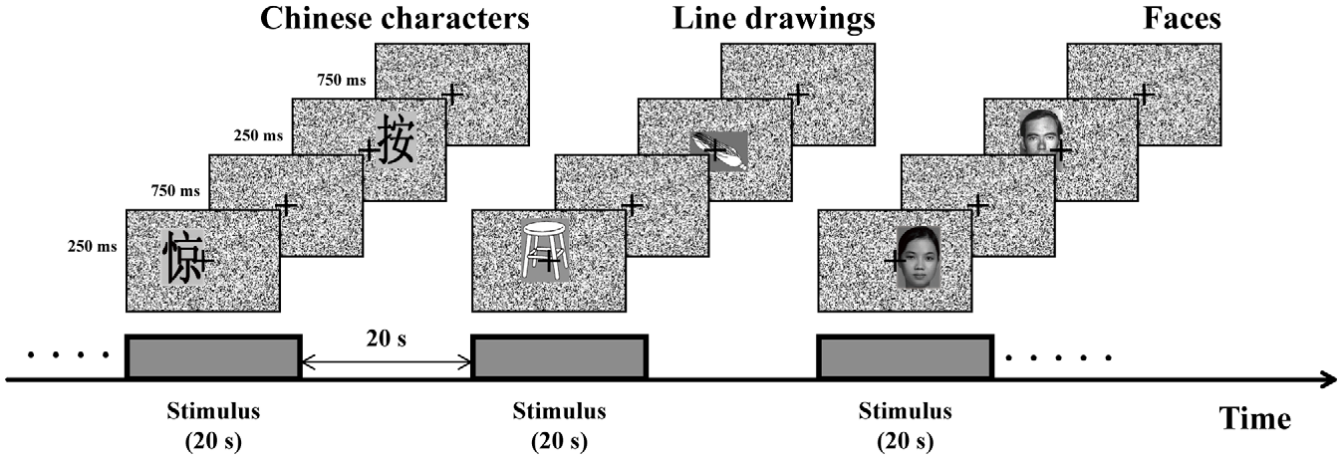
Schematic depiction of the experimental paradigm. Participants viewed Chinese characters (and native English speakers viewed English words in addition), faces, and line drawings in block-design runs with each block consisting of 20 stimuli.

In Exp. 1, native Chinese readers viewed Chinese characters, line drawings, and non-famous Chinese faces in block-design runs with each block consisting of 20 stimuli. Each stimulus was presented for 250 ms with an inter-stimulus interval (ITI) of 750 ms (Figure 1). Each run contained 9 blocks with 3 blocks for each category of stimuli. Within each block, the center of each stimulus was slightly shifted from the center of the fixation point, and participants were asked to make a judgment about whether the center of the picture was to the left or the right compared with the fixation point and pressed the corresponding buttons (left or right) as soon as possible. In Exp. 2, participants viewed the same three categories of stimuli (Chinese characters, faces, and line drawing objects) but performed either silent naming/reading task or position judgment task in separate blocks. The position judgment task was essentially the same as Exp. 1, while silent naming/reading task required subjects to silently name faces and line drawing stimuli or silently read Chinese characters after each stimulus presentation. For this reason, the face stimuli were all famous faces (politicians and actors). In order to check if subjects were indeed paying attention and performing the task, catch trials of unrecognizable objects, unfamiliar faces, and non-characters were included, and subjects were required to press a button to indicate any unnamable or unreadable stimuli.

### fMRI data acquisition and analysis

fMRI data were collected on a 3T Siemens Trio scanner at the Center for Magnetic Resonance Research (CMRR) at the University of Minnesota. Functional images were acquired with an EPI sequence with standard parameters (20 slices approximately parallel to the base of the temporal lobe, 3.0 mm slice thickness with no gap; field of view, 220×220 mm^2^; matrix, 64×64; repetition time, TR, 2000 ms; echo time, TE, 35 ms; flip angle, 75°). For each subject, a T1-weighted anatomical volume (3D MPRAGE; 1×1×1 mm^3^ resolution) was acquired for localization and visualization of the functional data.

Data were analyzed with BrainVoyager QX (Brain Innovation) software. After preprocessing (slice timing correction, 3D motion correction and temporal filtering), the functional data were coregistered with the anatomical data. Statistic maps of the brain were computed by performing general linear model multiple regression tests and ROIs were detected at the individual level, but group analyses were performed in a common Talairach space across subjects. fMRI responses to each category of stimuli were extracted and imported into MATLAB for further analyses. To highlight the differential contributions of different stimuli in the median, center, and lateral part of the mid-fusiform cortex, we proceeded to obtain a full response profile across the mid-fusiform surface for these stimuli. To do that, we first divided the current data set into even runs and odd runs, and used half of the data set (odd runs) to localize the ROIs (i.e., FFA), and a band of cortical surface (gray matters, width of two voxels) was selected that runs across the mid fusiform gyrus between the median to lateral part, intersecting the peak response location of the face stimuli and spanning around 40 mm. Next, from the other half of the data set (even runs), beta values of hemodynamic response fitting for the three categories of stimuli were extracted for each position. To obtain the average beta distribution curves, the mean distances between the peaks of each category of stimuli were calculated, and each individual subject's beta distributions were re-sampled according to these distances. Furthermore, the spatial profiles were aligned according to the peak position of the FFA before group averaging so that the tuning specificity was not severely broadened due to the anatomical variability across subjects.

## Results

To investigate the category selectivity for Chinese characters in the mid-fusiform cortex, we compared visual processing of Chinese characters with those of faces and line drawing objects, especially emphasizing very detailed activation patterns (Exp. 1). The reason for contrasting Chinese characters with faces and line drawings is that these stimuli, like characters, are complex visual stimuli and faces in particular have the best category selectivity in the mid-fusiform area (FFA, [26]) and can serve as a good landmark for the category activation distribution analysis. To further test the visual word form specialization in the mid-fusiform cortex and investigate the potential top-down modulation of phonological and/or semantic processing on category activation distribution, we compared distributions of voxel activity when observers performed silent naming/reading with when they did passive position judgment tasks for all these test stimuli (Exp. 2).

### Experiment 1: Details of category selectivity for Chinese characters in the left mid-fusiform cortex

**Figure 2:**
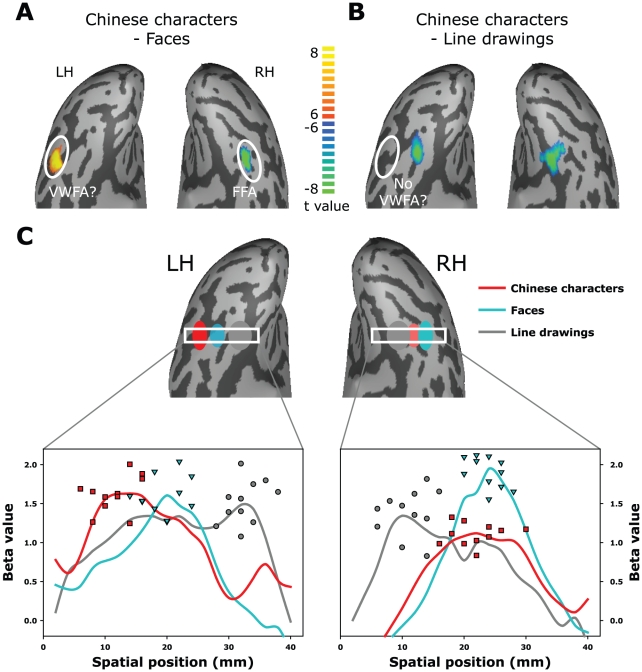
fMRI activation maps from native Chinese subjects who viewed Chinese characters, faces and line drawing objects. Results are displayed on a single subject's ventral occipital temporal cortical surface. **A**. Contrasting between characters and faces revealed a region in the lateral part of the left fusiform cortex with enhanced activation to Chinese characters (*p*<10^−6^, confirmed with Bonferroni correction, *p*(Bonf)<.05). This region is spatially consistent with the reported locations of the VWFA. A region with higher activity to faces was revealed in the right fusiform cortex, corresponding to the FFA. **B**. When contrasted with line drawings, the presumed VWFA no longer consistently shows a higher activation to Chinese characters. **C**. Beta values for voxels along a strip of cortex that covers both VWFA and FFA were first derived from a general linear model analysis for the three stimulus categories and then averaged across subjects. The resulting beta distributions for the three stimulus categories are plotted as functions of horizontal (X) positions, and the highest selectivity for Chinese characters lies in or near the occipito-temporal sulcus, lateral to the face sensitive region in the left mid-fusiform cortex. The small square, triangle, and circle symbols show the spatial positions as well as the beta values of the distribution peaks of the character, face, and line drawing activations respectively, for all individual subjects.

In a linear regression model, when activation from three categories of stimuli (Chinese characters, faces and line drawing objects) are contrasted with each other, the results show that a region in the right mid-fusiform (presumably rFFA: x/y/z = 38/-50/-15, *p*<10^−6^, confirmed with Bonferroni correction, *p*(Bonf)<.05) was consistently more strongly activated by faces than characters and line drawings, while line drawings consistently activated the medial part of the bilateral fusiform cortex more strongly than the other two categories(*p*<10^−6^ and *p*(Bonf)<.05). Moreover, enhanced activity to Chinese characters compared to faces could be seen in a region in the left mid-fusiform cortex (presumably VWFA: x/y/z = -38/-49/-12, *p*<10^−6^ and *p*(Bonf)<.05; see Fig. 2A). These observations are consistent with what have been reported before [27], [28]. However, at the group level, the area that showed higher activation to characters than faces was not more responsive to characters when compared to line drawings (Fig. 2B).

Since the random effect analysis was based on the spatially normalized data from all subjects and there was potential variability in the anatomical details of the cortical surface structure across individuals, we therefore analyzed the detailed spatial response profiles across the mid-fusiform surface for all stimulus categories. To do that, a band of cortical surface (gray matter, width of 2 mm) was selected that runs across the mid-fusiform gyrus between the medial and lateral parts, with the location of the peak response to the face stimuli as a landmark. Next, beta values of hemodynamic response fitting for the three categories of stimuli (Chinese characters, faces, and line drawings of objects) were extracted at each spatial position, and the distributions of beta-values were obtained for each individual subject.

In order to further obtain the group averaging beta distribution curves while avoiding the anatomical variability, each individual subject's beta distributions for each category of stimuli were first re-sampled with respect to their mean peak distances, and then the spatial profiles were aligned according to the peak position of the FFA before group averaging (see Methods for more detail). This procedure was performed so that the averaged beta curves from individual subjects would not be too severely broadened. The beta distributions from the 11 subjects were then averaged and plotted as the three curves in Fig. 2C for each hemisphere. The plots highlight the key properties of the mid-fusiform response to the three different object categories – selective response with spatially overlapped tuning. More specifically, it could be seen that 1) the most responsive region for line drawings of regular objects tends to be more distributed and lie in the medial part of the bilateral fusiform gyri (gray curves and gray symbols); 2) the response for faces tends to peak in the middle/lateral part of the fusiform gyrus with the right FFA showing a more prominent and sharply peaked response than the left (cyan curves and cyan symbols; amplitudes of peak responses (RH vs. LH): t(10) = 4.02, *p*<0.003;); 3) responses to Chinese characters are more significant in the left mid-fusiform region than the right (red curves and red symbols; amplitudes of peak responses (LH vs. RH): t(10) = 4.91, *p*<0.001), and the highest selectivity for Chinese characters tends to occur lateral to the face sensitive region and often lie in or near the left occipito-temporal sulcus (locations of peak responses (VWFA vs. FFA): t(10) = 5.98, *p*<0.001). The peak beta values for the three categories of all subjects form three statistically distinct clusters, especially in the left hemisphere (see the three types of symbols in Fig. 2C; repeated measures analysis of variance (ANOVA): F(2,30) = 132, *p*<0.001).

The results clearly show category selectivity in the mid-fusiform cortex for Chinese characters. Although the responses to the three categories of stimuli were spatially overlapping with each other, the responses' peaks are clearly separated for Chinese characters compared to other visual stimuli. It is also worth noting that, in terms of a simple measure of response amplitudes, even in the presumed VWFA, Chinese characters were not necessarily the highest in all subjects. We suspect that this as well as the anatomical variability across subjects may be the reasons that some studies failed to reveal the specialization for words compared with line drawings in the mid-fusiform cortex [14], [15]. Nevertheless, when we look at the distribution of response patterns across written scripts and the other categories of stimuli (Fig. 2C), the segregation between categories becomes very clear. We suggest that the response amplitude in a predefined ROI (e.g., VWFA) could be modulated by many factors such as task demand, whereas the overall pattern of activation constitutes a better indicator of specialization. The pattern of specialization shown in Figure 2C will be further explored in terms of task modulation (Exp. 2).

### Experiment 2: Category selectivity for Chinese characters independent of task modulation

Exp. 1 clearly demonstrated a distinct pattern of activation in the left mid-fusiform cortex for Chinese characters, with a lateral bias compared to that for faces and line drawing objects. This pattern of activation is consistent with the notion that the VWFA is specialized for written words (in this case Chinese characters). However, this result was obtained when subjects performed a content-irrelevant task (judging whether the stimulus was slightly to the left or right of fixation), and it remains possible that the laterally biased activation for characters is influenced by task demands. In other words, the lateral part of the left mid-fusiform may not be specialized for input category (written scripts) per se, but rather serves as a functional interface between the visual structural encoding and high level phonological and semantic information [18], [29], [30], [31], the lateralized activation for characters might be due to the automatic phonological and semantic encoding of characters compared to the other categories of stimuli in the passive (content-irrelevant) task. This issue is very difficult to resolve with a traditional BOLD amplitude in a restricted ROI approach, because it is hard to predict how much attentional and task modulation effect is expected for each category of stimuli. In contrast, the patterns of activation revealed in Exp. 1 for different stimuli categories allow for a better test of the category selectivity and task/attentional modulation. If the characters and line drawings do not have intrinsic distinct patterns of activation (e.g., VWFA is lateral while line drawings are more distributed) and the lateralized activation of characters shown in Fig. 2C was due to their automatic phonological and semantic processing, then an explicit and demanding phonological and semantic task would result in overall lateral shift of activation distributions for all categories of stimuli in the left mid-fusiform area. On the other hand, if the distinct activation distribution pattern preserves and is independent from the task modulation, despite a higher or lower response magnitude due to task modulation or attentional effect, it will provide strong support that there is a specialized region for processing Chinese character form information and the categorical activation distribution may be a better indicator compared with traditional group or ROI analysis. Simply, the current question becomes whether the pattern of activation would shift laterally or not depending on the task demand.

Therefore, we tested this issue explicitly in this experiment by adopting a silent naming/reading task. The same three categories of stimuli (Chinese characters, faces, and line drawing objects) were used in this experiment, and subjects were asked to perform a silent naming (for faces and line drawing stimuli) and silent reading (for Chinese characters) task after each stimulus presentation. For this reason, the face stimuli were all famous faces (politicians and actors). The silent naming/reading task was adopted to push the processing of faces and line drawing stimuli to the phonological and semantic level but still minimize subjects' head motion. In order to check if subjects were indeed paying attention and performing the task, catch trials of unrecognizable objects, unfamiliar faces, and non-characters were included, and subjects were required to press a button to indicate any unnamable or unreadable stimuli. The passive position judgment task (the same as Exp. 1) was run in separate blocks so that the potential task modulation of the visual word form specialization could be directly examined.

Results from ROI analysis clearly showed a task modulation of the response magnitudes. There were stronger fMRI responses to all three categories of stimuli (relative to the fixation baseline) in the active naming/reading task compared with the passive position judgment task (F(1,6) = 12.0, *p*<0.02) due to the demanding task. More specifically, although the presumed VWFA could be consistently identified in the left mid-fusiform region with a contrast between Chinese characters and face stimuli in the passive task (t(6) = 4.64, *p*<0.005, consistent with Exp. 1), the same areas didn't show a significantly higher response to Chinese characters than face or line drawing stimuli in the active task (*p*>0.3), consistent with previous findings [18], [19]. Therefore, results from the traditional ROI analysis seemingly provided support for the argument against the special role of the VWFA in processing word form. However, the critical test came next regarding the spatial distributions of the activation under the two tasks.

**Figure 3:**
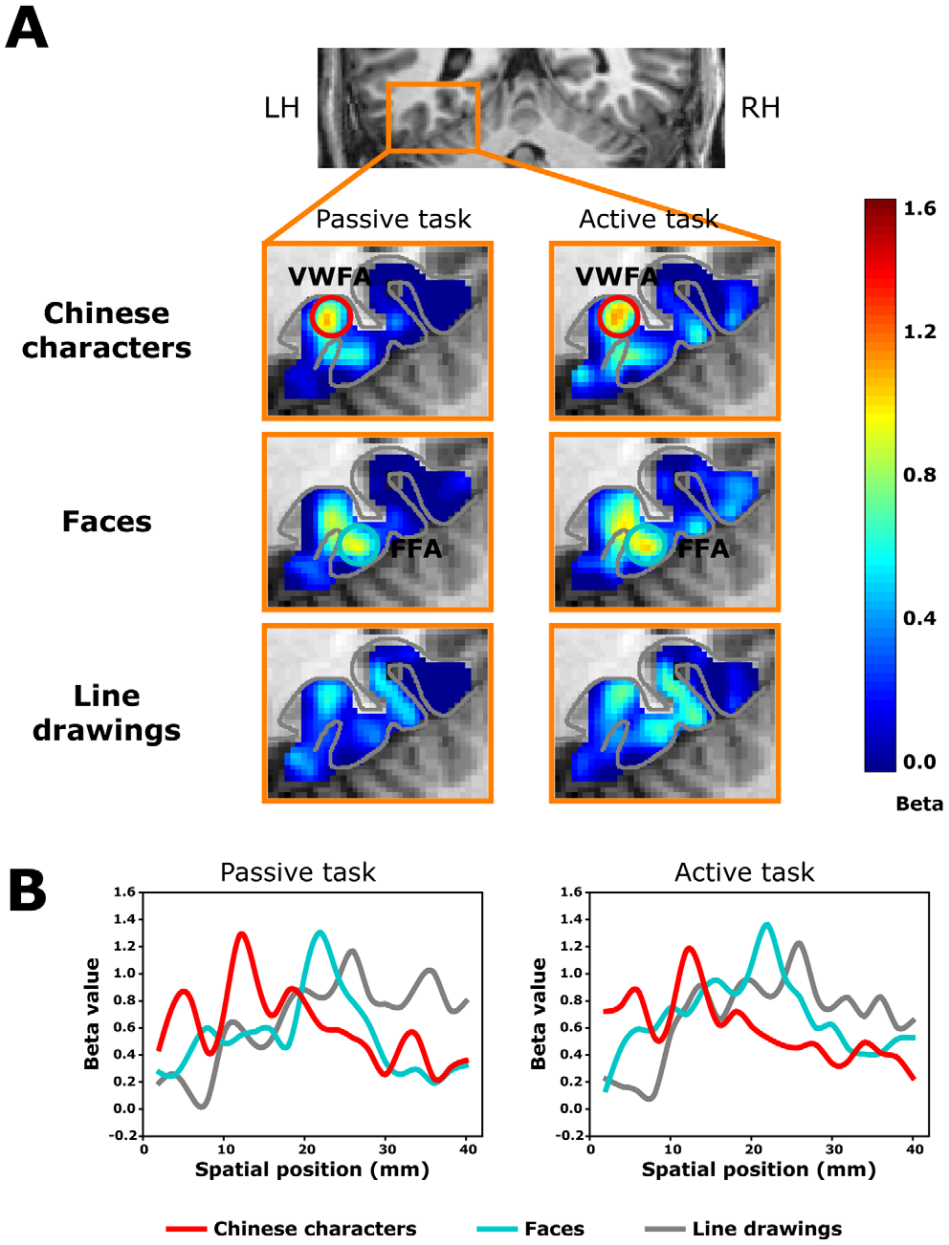
Spatial profile of response selectivity across mid-fusiform cortex in the active (naming/reading) and passive (position judgment) tasks. Beta distributions were extracted from the grey matter surface along the mid-fusiform cortex and were plotted as a function of spatial position. The anatomical images were of a single subject (A) and the beta distributions were averaged from all subjects (B). The distributions for the three categories of stimuli (Chinese characters, faces, and line drawings) were similar in the passive task (left panels) and the active task (right panels), supporting a robust and task-independent response selectivity for Chinese characters in the left lateral mid-fusiform cortex.

**Figure 4:**
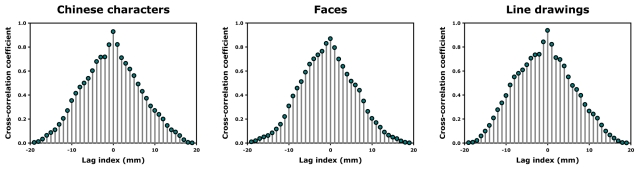
Cross-correlation coefficients of beta values as a function of spatial position between the two tasks (passive vs. active). For each category of the test stimuli, the beta distributions were not shifted from the passive task to the active task (peak correlation at zero lag), and the average correlation coefficient of the zero lag was 0.92±0.06, indicating that neither the peak positions nor the shapes of the beta distributions were changed between these two tasks.

Thus we extracted the beta value distributions in the mid-fusiform region for the three categories of stimuli for each subject, similar to Exp. 1, but in both the active (naming/reading) and passive (position judgment) tasks. Consistently, in the passive task, we found a lateralized peak for Chinese characters in the left mid-fusiform cortex whereas the faces and line drawing objects were peaked in the medial and middle parts of the left mid-fusiform, respectively (Fig. 3, left panels). This pattern of results replicated what was found in Exp. 1. Critically, as can be seen from Fig. 3, the beta distributions for the three categories of stimuli (Chinese characters, faces, and line drawing objects) were essentially the same in both the silent naming/reading (Fig. 3, right panels) and the position judgment tasks. To statistically test if there is any potential peak shifts in terms of activation distributions, we computed the cross-correlations of beta values as a function of spatial position between these two tasks (passive vs. active) for each category of the test stimuli. This measure is effective in detecting both the peak shifts (indexed by lag indices) and the shape changes (indexed by correlation coefficients) of the beta distributions (see Methods for details). The lag indices and their respective correlation coefficients for each category were shown in Figure 4. Clearly, the beta distributions for all three categories of stimuli were not shifted from the passive task to the active task (lag indices: t(6) = 1.22, *p*>0.2), and the average correlation coefficient of the zero lag (no peak shift) between the two tasks was 0.92±0.06, indicating that neither the peak positions nor the shapes of the beta distributions were changed between these two tasks (despite a significant task modulation of the response magnitude).

## Discussion

Using activation distribution analysis, a region in the left mid-fusiform cortex could be consistently identified that showed a selective response pattern to Chinese characters when compared to faces or line drawings of objects. This region, presumably the VWFA, was specialized for processing visual word/character form information. However, the category selectivity of the VWFA was not simply revealed in the averaged response amplitude levels using a traditional ROI approach [17]. Rather, there is a unique and robust spatial pattern of activity in this region that allows the Chinese characters to be distinguished from other categories of visual input (e.g., faces and line drawings of common objects). In addition, the specialized activation distribution of Chinese characters in the left mid-fusiform region is independent of task modulation, thus the unique pattern of activation constitutes a better indicator of specialization than the traditional group and ROI-based analyses.

Our results provide a comprehensive picture of the response properties of the mid-fusiform cortex. There is clear evidence for functional specialization within this region, but looking through the peaks of functional selectivity, we also see relatively broad and variable response distributions across subjects due to anatomic variability. The consequence of this relatively broad distribution of activation is that if a group analysis is adopted or an inappropriate ROI is defined within this area [32], the ROI would most likely show response to a wide spectrum of visual stimuli, with the amplitude of response less reliable as an indicator of the ROI's selectivity. Indeed, the variability in the predefined VWFA's response amplitude (modulated by task, stimulus selection, visual training, etc) is partly responsible for the divergent views about the functional selectivity of the VWFA. We believe our study provided a more stable functional characterization for the VWFA with the spatially distributed pattern of activation.

Although the selectivity for word form in the VWFA is not as robust as for faces in the FFA, we could see that spatial and functional selectivity for written scripts in the ventral occipito-temporal cortex have some commonalities compared with the selectivity for faces. This is interesting given that there is a very strong and long history of evolutionary pressure for face selectivity, but very short history and much weaker (if any) evolutionary pressure on reading. The firm establishment of a specialized processing region for written scripts provides a unique opportunity for more extensive future research on the genesis of cortical specialization.

### Conclusion

Our study provides strong and clear evidence for functional specialization for visual processing of written scripts in the human ventral occipito-temporal cortex. In normal readers a region in the lateral part of the left mid-fusiform gyrus, lateral and adjacent to the left FFA, shows robust specialization for distinctive orthographic units (e.g., Chinese characters for Chinese readers). This category selective response is clearly revealed in the distinct patterns of response, compared to traditional group and ROI analyses. The distinct patterns of response to the different stimulus categories were independent of task modulation.
